# Challenges and Opportunities in Pathogen Agnostic Sequencing for Public Health Surveillance: Lessons Learned From the Global Emerging Infections Surveillance Program

**DOI:** 10.1089/hs.2023.0068

**Published:** 2024-02-19

**Authors:** Lindsay Morton, Kathleen Creppage, Nazia Rahman, June Early, Laurie Hartman, Ashley Hydrick, Matthew Kasper

**Affiliations:** Lindsay Morton, MPH, MS, is a Senior Molecular Epidemiologist; GEIS Branch, Armed Forces Health Surveillance Division, Defense Health Agency, Silver Spring, MD.; Kathleen Creppage, DrPH, MPH, is a Scientific Program Manager and Technical Lead; GEIS Branch, Armed Forces Health Surveillance Division, Defense Health Agency, Silver Spring, MD.; Nazia Rahman, MPH, is a Molecular Epidemiologist and Portfolio Manager; GEIS Branch, Armed Forces Health Surveillance Division, Defense Health Agency, Silver Spring, MD.; June Early, MPH, is Global Emerging Infections Surveillance (GEIS) Deputy Chief; GEIS Branch, Armed Forces Health Surveillance Division, Defense Health Agency, Silver Spring, MD.; Laurie Hartman, MS, is a former Laboratory Support Specialist; GEIS Branch, Armed Forces Health Surveillance Division, Defense Health Agency, Silver Spring, MD.; Ashley Hydrick, DVM, MPH, is a Major, US Army, and former GEIS Focus Area Chief; GEIS Branch, Armed Forces Health Surveillance Division, Defense Health Agency, Silver Spring, MD.; Matthew Kasper, PhD, is a Commander, US Navy, and GEIS Chief; GEIS Branch, Armed Forces Health Surveillance Division, Defense Health Agency, Silver Spring, MD.

**Keywords:** Sequencing, Pathogen agnostic, Metagenomics, Public health preparedness/response, Surveillance, Infectious disease

## Introduction

Pathogen agnostic sequencing has been identified as a tool for biodefense and public health given its role in infectious disease detection and monitoring. US governments interest in this tool is apparent in the most recent US National Biodefense Strategy,^1^ the American Pandemic Preparedness Plan,^2^ and fiscal year 2023 Broad Agency Announcements from the US Department of Health and Human Services, including the US Centers for Disease Control and Prevention (CDC) and Biomedical Advanced Research and Development Authority.

Next-generation sequencing (NGS) technologies, which are higher-throughput genome sequencing technologies developed after Sanger sequencing and can be used for pathogen agnostic sequencing, have become more affordable and more widely adopted among biomedical and public health laboratories.^3^ Furthermore, the cost per genome generated or samples sequenced has decreased dramatically, accelerating the transition of NGS from a research activity to an essential component of routine public health surveillance.^4,5^

Pathogen agnostic sequencing—also referred to as metagenomic, shotgun, unbiased, or random-priming sequencing—is typically conducted when more information is desired about the microbial community within a sample or when more specific assays, such as polymerase chain reaction or amplicon sequencing, fail. These assays may fail due to suboptimal design or selection, poor or missing reference genomes, the evolution of target pathogens, or the emergence of a novel or divergent human pathogen.^3^ Recent approaches to pathogen agnostic sequencing have focused on clinical metagenomics, primarily for patient diagnostics, discovery of novel or unexpected human pathogens, and screening of environmental samples. Some of the earliest advances in routine pathogen agnostic sequencing were made within the field of clinical metagenomics for the detection of unknown pathogens in patient samples to determine the likely cause of underlying illness or mortality.^6^ Within public health, pathogen agnostic sequencing approaches have long been used in surveillance for novel and emerging animal and human pathogens with risk for spillover or pandemic potential.^7,8^ This can be a time- and resource-intensive “needle in the haystack” approach, depending on the sample source, collection method, and preservation method. Additionally, local capacity (eg, equipment and information technology infrastructure), interorganizational data-sharing agreements, and capabilities (eg, well-trained laboratories and subject matter experts) are necessary for the development, implementation, and maintenance of a pathogen agnostic sequencing program.

While pathogen agnostic sequencing technologies and testing approaches have matured and become more accessible, fewer considerations have been made for the operationalization of agnostic sequencing within the context of an infectious disease surveillance system, such as the Global Emerging Infections Surveillance (GEIS) program of the US Department of Defense (DOD). Given widespread access to sequencing technologies, longstanding surveillance studies with ongoing sample collections and repositories, and robust expertise in NGS and bioinformatics among DOD scientists, the GEIS program is well positioned to implement, maintain, and generate meaningful information from pathogen agnostic sequencing within its surveillance network. We believe the DOD GEIS program has gained experience in several important lessons related to the operationalization and implementation of pathogen agnostic sequencing within an existing infectious disease surveillance program. The purpose of this commentary is to use an existing DOD infectious disease surveillance program to highlight opportunities and challenges for the implementation of pathogen agnostic sequencing in the context of the wider global community.

## History of Pathogen Agnostic Sequencing in the GEIS Program

The GEIS program operates through a global network of US Army, Navy, and Air Force medical research, clinical, and public health laboratory partners in strategic locations, which are engaged on the front lines of global infectious disease surveillance and have longstanding relationships with the US interagency, host-nation, and international partners.^9,10^ These partners share laboratory-confirmed infectious disease surveillance data within the network, some of which may be indicative of potential emerging threats to US service members. Epidemiological and clinical metadata may also be available due to the existence of robust sentinel site programs and standardized, coordinated data collection processes. This serves to enrich the “picture” of an infectious disease pathogen, connecting etiology, transmission, and clinical outcomes to genomic characterization. However, this reporting is not done in real time. While GEIS-funded partners may facilitate rapid communications on the ground with local organizations if needed, they are required to submit data on a monthly basis, at minimum, to the GEIS program office. When high- or moderate-level threats to force health protection are identified, partners must submit reports in as quickly as 24 hours to the GEIS program office and the geographic combatant commands to inform their decisionmaking.

The GEIS program leveraged early DOD biodefense and medical research investments in pathogen sequencing, made in 2014-2015, to enhance infectious disease surveillance activities performed by GEIS partner laboratories. Some of these critical investments included the Global Biosurveillance Technology Initiative operated by the Joint Program Executive Office for Chemical and Biological Defense (now the Joint Program Executive Office for Chemical, Biological, Radiological, and Nuclear Defense), which delivered Illumina MiSeq instruments, ancillary library preparation equipment, and bioinformatics servers to DOD laboratories located throughout the United States and abroad.^11,12^ These efforts were implemented partly in response to priorities indicated in the 2012 *National Strategy for Biosurveillance*,^13^ which included building a coordinated, integrated biosurveillance infrastructure.

**Figure 1.**
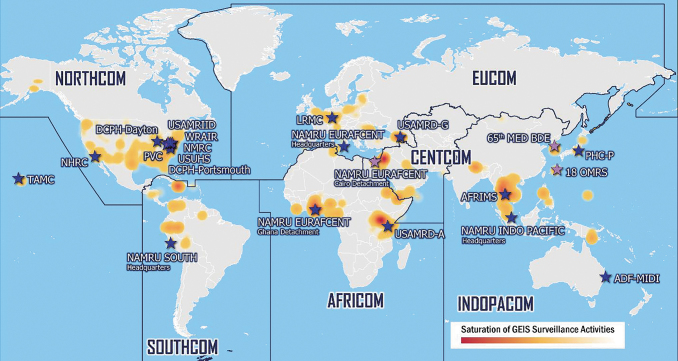
GEIS surveillance activities and GEIS partner laboratories with next-generation sequencing equipment (denoted with dark blue stars). These DOD medical research, clinical, and public health laboratories are operated by the US Army, US Navy, US Air Force, and Defense Health Agency, and include host-nation partners in select locations worldwide. Abbreviations: 18 OMRS, 18^th^ Operational Medical Readiness Squadron; 65^th^ MED BDE, 65^th^ Medical Brigade; ADF-MIDI, Australian Defence Force Malaria and Infectious Disease Institute; AFRICOM, Africa Command; AFRIMS, Armed Forces Research Institute of Medical Sciences; CENTCOM, Central Command; DCPH-Dayton, Defense Centers for Public Health Dayton; DCPH-Portsmouth, Defense Centers for Public Health Portsmouth; DOD, US Department of Defense; EUCOM, European Command; GEIS, Global Emerging Infections Surveillance; INDOPACOM, Indo-Pacific Command; LRMC, Landstuhl Regional Medical Center; NAMRU INDO PACIFIC, Naval Medical Research Unit Indo Pacific; NAMRU EURAFCENT, Naval Medical Research Unit supporting AFRICOM, CENTCOM, and EUCOM; NAMRU SOUTH, Naval Medical Research Unit South; NECE, Navy Entomology Center of Excellence; NHRC, Naval Health Research Center; NMRC, Naval Medical Research Command; NORTHCOM, Northern Command; PHC-P, Public Health Command-Pacific; PVC, Pharmacovigilance Center; SOUTHCOM, Southern Command; TAMC, Tripler Army Medical Center; USAMRD-A, US Army Medical Research Directorate-Africa; USAMRD-G, US Army Medical Research Directorate-Georgia; USAMRIID, US Army Medical Research Institute of Infectious Diseases; USUHS; Uniformed Services University of Health Sciences; WARUN, Walter Reed AFRIMS Research Unit Nepal; WRAIR, Walter Reed Army Institute of Research.

**Figure 2.**
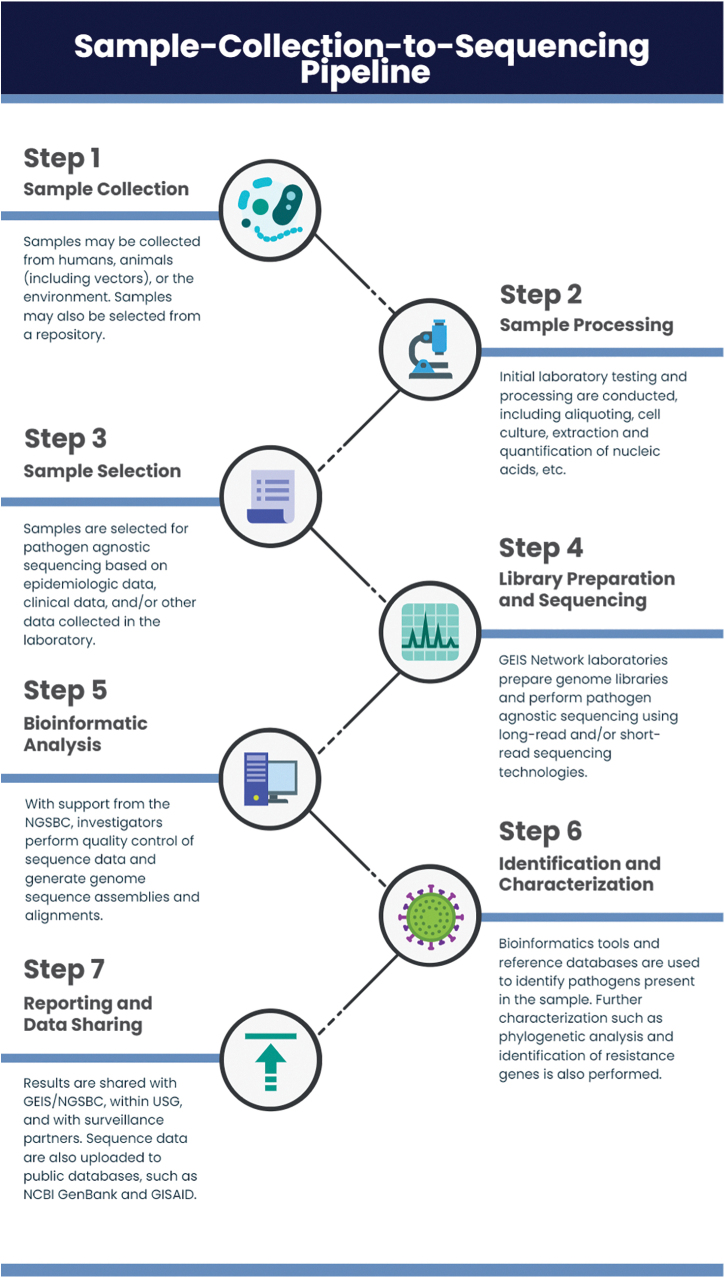
Sample collection and processing workflow for pathogen agnostic genomic surveillance. Abbreviations: GEIS, Global Emerging Infections Surveillance; GISAID, Global Initiative on Sharing All Influenza Data; NCBI, National Center for Biotechnology Information; NGSBC, Next Generation Sequencing and Bioinformatics Consortium; USG, US government.

Pathogen agnostic sequencing gradually integrated into the GEIS program over the past decade as NGS technologies became more widespread among DOD laboratories. Currently, GEIS partners with pathogen agnostic sequencing capabilities are located across North America, Europe, South America, Africa, Southeast Asia, and the Pacific (Figure 1). These laboratory partners are also actively engaged in prospective sample collection, maintenance of pathogen repositories, and studies supporting infectious disease surveillance in the United States and abroad.^14-16^ Within the GEIS network, a concerted effort is required to harmonize activities from sample collection to sequencing. The sample-collection-to-sequencing pipeline progresses as follows: (1) sample collection from the field or a repository, (2) initial sample processing and testing, (3) samples selection for pathogen agnostic sequencing, (4) performance of wet laboratory sequencing, (5) bioinformatics analysis, (6) generation of actionable sample information, and (7) data sharing (Figure 2). Epidemiological data are often collected in parallel with sample collection through a survey instrument or medical record, although the quality and quantity of this information varies throughout the network. In more resource-constrained environments, it can be challenging to integrate sequencing outcomes with metadata depending on how data are collected and stored.

## Implementation of Pathogen Agnostic Sequencing Within the GEIS Network

**Figure 3.**
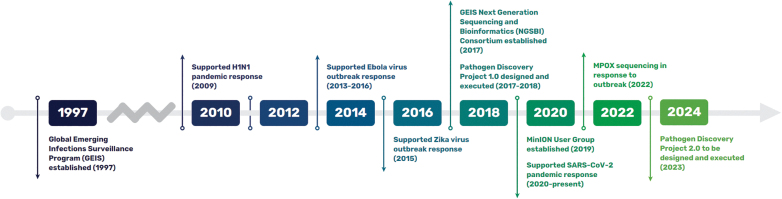
Global Emerging Infections Surveillance Program history and pathogen sequencing milestones. Abbreviation: GEIS, Global Emerging Infections Surveillance.

The GEIS program has implemented several initiatives to use and harmonize pathogen agnostic sequencing within an existing infectious disease surveillance program (Figure 3).

### Next Generation Sequencing and Bioinformatics Consortium

In 2017, the GEIS program established the Next Generation Sequencing and Bioinformatics Consortium, composed of pathogen genome sequencing and bioinformatics experts from DOD medical research laboratories around the world, as shown in Figure 1. This consortium works to promote collaboration, communication, and standardization for NGS and bioinformatics practices among the GEIS network of DOD laboratory partners, with the goal of increasing the availability, utility, and quality of pathogen sequence data. These data feed into timely information products to inform force health protection and DOD public health efforts domestically and worldwide. Furthermore, in 2019, the consortium established a working group of mobile NGS users to provide a forum for discussing and troubleshooting newer technologies, demonstrating a commitment to evolve and respond to both emerging infectious diseases and emerging technologies for pathogen detection and characterization.

### Pathogen “Discovery” Project 1.0

To initially guide GEIS pathogen-sequencing activities, the consortium conducted 2 major efforts to better understand sequencing capabilities and gaps within the network. First, the consortium surveyed GEIS partner laboratories in 2017 to understand current NGS and bioinformatics capabilities, areas of interest, and strategic needs. This was followed by a proficiency testing exercise, referred to as the Pathogen Discovery Project 1.0, in which participating laboratories received a blinded panel of human pathogens in various matrixes and tested it using their existing NGS protocols. Results showed that most laboratories used an agnostic (or metagenomic) sequencing approach. The raw data were used to evaluate laboratory pathogen detection and characterization capabilities. This exercise led to the development of training modules to address gaps, including modules for optimizing metagenomic sequencing and bioinformatics analysis.^17^

### GEIS-Funded Surveillance Activities Using Pathogen Agnostic Sequencing, Including Early Outbreak Response

Historically, the GEIS program has most frequently supported pathogen agnostic sequencing activities for surveillance projects across its respiratory and febrile and vector-borne infections portfolios. Prior to the COVID-19 pandemic, the GEIS program prioritized “pathogen-negative” samples from routine surveillance activities for agnostic sequencing, focusing on cases of acute respiratory infections or acute febrile illnesses that failed to yield a result using traditional diagnostics.^18^ Additionally, several instances of pathogen agnostic methods have been used for arbovirus characterization^19,20^ or for pathogen discovery in vector samples.^21-23^ Although not strictly “agnostic,” GEIS partner laboratories have also developed or used sequencing methods that improve upon traditional metagenomic methods and integrate optimization steps for host depletion or viral pathogen enrichment.^24,25^ In early outbreak or pandemic responses, pathogen agnostic or panviral sequencing methods were used to characterize Zika virus, SARS-CoV-2, and mpox cases within the GEIS network, before numerous reference genomes were available or standard amplicon-based sequencing protocols were widely adopted.^20,26^ However, as illustrated in several of these examples, targeted sequencing or enrichment is often used in conjunction with pathogen agnostic methods to confirm findings or generate higher-quality genomes.

## Lessons From the GEIS Program

Challenges with operationalizing pathogen agnostic sequencing have been well documented.^6,27-29^ The GEIS program has firsthand experience with the challenges associated with deploying this method for general biosurveillance, from difficulties with implementation and policy development to technical issues surrounding information technology access and requirements (Box).

**Box. Challenges to Operationalizing a Pathogen Agnostic Surveillance System**

1. Identification of appropriate use cases/sample sets
2. Rapidly advancing or changing sequencing and bioinformatics technologies/methods
3. Lack of community standards
4. Reproducibility of results
5. Establishment of data- and sample-sharing agreements with partner laboratories and host nations
6. Staffing recruitment, retention, and training
7. Supply chain and equipment servicing (particularly for low- or middle-income countries)
8. Access to computational resources, information technology staff support, and updated reference databases
9. Access to stable funding sources for public health surveillance activities
10. Integration with existing clinical or public health surveillance systems
11. Policy gaps for the use of pathogen agnostic sequencing results for public health action

While expertise in the field related to pathogen agnostic methods is abundant, it can be challenging to recruit, retain, and mentor highly trained molecular epidemiologists, laboratorians, and bioinformaticians (particularly in resource-constrained areas).^28^ Supporting up-to-date training for more complex methods associated with pathogen agnostic sequencing can also be challenging, especially when demands for known pathogen targets (eg, SARS-CoV-2) become prioritized. It can also be extremely difficult to acquire or access sufficient supplies, computing power, and well-maintained databases in resource-limited settings.^28^

One of the most important components of conducting these activities across a broad network with a mixture of stakeholders is establishing necessary agreements and ensuring they are in place for current and future needs. These agreements (whether for data use, sample sharing, or other purposes) are vital to protect the interests of host nations, the US military service laboratories, and other supporting organizations. Even within the GEIS network, the establishment of agreements for sample sharing and data sharing among partners is a complex and time-consuming process. With respect to pathogen agnostic sequencing, the outcomes of pathogen agnostic sequencing could have enormous public health implications for all stakeholders. Having agreements in place is particularly important if there is sensitivity around potential threats that could be detected or for the transport of potentially hazardous samples.

There is a general lack of standardization and regulation across the scientific community regarding protocols and agreed-upon methods for pathogen identification and characterization using agnostic sequencing.^30^ Given the rapid evolution of these technologies and techniques, it is not unexpected to see laboratories use a wide variety of kits, equipment, sample selection criteria, data collection tools and processes, and analytic programs. Even within the GEIS network, similar activities may not incorporate standard, harmonized sample selection criteria to achieve similar ends. This can lead to challenges with reproducibility, compounded by the complexity of technical issues such as sample cross-contamination, complex matrixes, and identifying appropriate thresholds.^6^

Similarly, there are mixed recommendations as to how to best use these technologies and their resulting outcomes to inform public health actions. Few, if any, policies exist that outline how to appropriately interpret, disseminate, and integrate findings from pathogen agnostic sequencing into existing public health systems and decisionmaking processes. One of the more challenging questions to address regarding pathogen agnostic methods is where they fit in the field of public health and emerging infectious disease surveillance, and when it is appropriate to implement them with their inherent risks. Pathogen agnostic sequencing is an informative adjunct to more traditional surveillance activities; without a priori knowledge of what a causative agent might be, it is difficult to use typical laboratory testing methods to characterize a potential emerging threat. Pathogen agnostic sequencing can provide valuable information on previously uncharacterized genomes, including critical mutations that might affect traditional diagnostic outcomes, which can then inform new diagnostic tools and techniques for future use. However, despite its many advantages, it is not always the most appropriate or cost-effective method. For instance, when targets are known and qualitative measures of detection are sufficient, less cost- and labor-intensive methodologies, such as polymerase chain reaction, can be used. There is also a potential risk in using these methods without reward; while pathogen agnostic methods can certainly have high value if and when they result in the detection of an unknown threat, the likelihood of this can vary depending on the epidemiology of the pathogen and the resulting clinical manifestation, the geographic location from which the sample was collected, and the skills and resources available at the laboratory to perform these more challenging techniques and interpret the findings.

## Opportunities for GEIS to Leverage Pathogen Agnostic Sequencing

The incorporation of sequencing capabilities into the GEIS partner laboratory network demonstrates the GEIS program's ability to integrate advanced laboratory methods into the program and ensure that laboratories remain at the forefront of pathogen detection and characterization. With renewed global interest in pandemic preparedness and novel approaches to infectious disease surveillance, the GEIS program has many opportunities to further develop and use pathogen agnostic sequencing:

1. Utilize and standardize testing algorithms that are not reliant on preexisting (biased) targeted or panel molecular tests
2. Support novel pathogen discovery among unique sample sets (eg, environmental, vector, human) collected by GEIS partners and to support development of new molecular assays
3. Provide more information for “pathogen-negative” samples derived from acute febrile illness and acute respiratory illness cases identified through GEIS surveillance activities
4. Access additional USG funding streams and maintain alignment with national and international biosurveillance and biodefense priorities
5. Maintain and expand early pandemic preparedness for emerging pathogens within the US DOD and among host-nation partners
6. Collaborate with and contribute to USG interagency and international pathogen agnostic sequencing surveillance systems (eg, wastewater surveillance)

## Discussion

The relationship between the biodefense and public health communities has evolved and likely been accelerated and enriched by the need for collaboration and communication since the onset of the COVID-19 pandemic.^31,32^ Regardless of how the surveillance activity is framed, both biodefense and public health perspectives are important components of overall biosurveillance activities. By monitoring infectious disease threats through pathogen agnostic approaches as part of large-scale biosurveillance activities across the DOD, the GEIS program can better prepare to (1) integrate future data and processes into existing DOD systems, much like the CDC Data Modernization Initiative and the US Department of Health and Human Services Trusted Exchange Framework and Common Agreement, (2) collaboratively make critical decisions about pandemic preparedness and response, (3) better understand the gaps in knowledge around emerging infectious threats, and (4) apply innovative and evolving technologies to improve DOD capability for novel pathogen identification and characterization.

Much of this is tied into modernizing DOD infrastructure and systems to move the needle toward standardized, integrated data and surveillance systems. While the CDC has developed an initiative that maps a broad, adaptable approach that can be applied to any organization for data modernization, the DOD has leaned in to begin integrating advanced technologies, such as artificial intelligence and cloud-based storage, into its existing systems. These efforts, which will ensure the accessibility of metadata across various domains, are currently underway; upon completion, they will be documented in a future publication. However, other requirements for a harmonized network approach to pathogen agnostic sequencing beyond data modernization include the following:

- Adequate, stable funding to increase and maintain equipment, information technology infrastructure, resources, personnel, and training to sustain activities over time
- A DOD-wide strategy and implementation plan for appropriate operationalization of pathogen agnostic sequencing for both routine surveillance and responses to potential public health emergencies, including coordinated scaling up during times of need
- Translation of funding for pathogen agnostic sequencing into robust, measurable, quality surveillance outcomes and force health protection measures within existing surveillance activities and infrastructure
- Data use and sharing agreements that clearly specify requirements around sample shipping, analysis support, and reporting of results

Maintaining a laboratory network that is capable and prepared to use pathogen agnostic sequencing methodologies to identify and characterize emerging pathogens is challenging. Capital investments must be continuously considered to ensure DOD laboratories remain at the forefront with current equipment. In addition, nothing is more critical than maintaining an elite workforce in a highly competitive talent marketplace. The COVID-19 pandemic has increased demand for public health professionals and scientists to develop clear plans to operationalize a coordinated response to threats detected through a pathogen agnostic surveillance system. The GEIS mission is force health protection, and continued investment in pathogen agnostic technologies offers an opportunity to enhance data products delivered by the GEIS partner network. Regardless of an emerging pathogen's origin, the GEIS program must continue to coordinate among partner laboratories to ensure and maintain a robust capability to detect and respond to infectious disease threats.

## Conclusion

Past DOD medical research and biodefense investments in NGS and pathogen agnostic testing have bolstered public health surveillance and paved the way for capabilities that will likely be critical for preparing and responding to the next pandemic.^33^ Since its establishment in 1998, the GEIS program has considered the biosurveillance landscape and invested in surveillance and advanced characterization infrastructure accordingly. These investments have continued to support the need for timely information on emerging infectious threats over time within the DOD, which maintains and expands such capabilities for wider public health surveillance. We believe these technologies are more impactful over the long term within a robust and coordinated laboratory network for infectious disease surveillance and response. While the GEIS program continues to incorporate pathogen agnostic sequencing into partner laboratories, a foundation exists upon which capabilities can rapidly scale up to respond to emerging infectious disease threats in support of health protection for US forces and general public health. Continued investment in these capabilities, from workforce and training to next-generation technologies, will be critical for defending against novel future threats.
